# Epigenetic modification in diabetic kidney disease

**DOI:** 10.3389/fendo.2023.1133970

**Published:** 2023-06-30

**Authors:** Zhe Liu, Jiahui Liu, Wanning Wang, Xingna An, Ling Luo, Dehai Yu, Weixia Sun

**Affiliations:** ^1^ Public Research Platform, First Hospital of Jilin University, Changchun, Jilin, China; ^2^ College of Basic Medical Sciences, Jilin University, Changchun, Jilin, China; ^3^ Department of Nephrology, First Hospital of Jilin University, Changchun, Jilin, China

**Keywords:** epigenetic modification, diabetic kidney disease, metabolic disorder, biomarker, noncoding RNA

## Abstract

Diabetic kidney disease (DKD) is a common microangiopathy in diabetic patients and the main cause of death in diabetic patients. The main manifestations of DKD are proteinuria and decreased renal filtration capacity. The glomerular filtration rate and urinary albumin level are two of the most important hallmarks of the progression of DKD. The classical treatment of DKD is controlling blood glucose and blood pressure. However, the commonly used clinical therapeutic strategies and the existing biomarkers only partially slow the progression of DKD and roughly predict disease progression. Therefore, novel therapeutic methods, targets and biomarkers are urgently needed to meet clinical requirements. In recent years, increasing attention has been given to the role of epigenetic modification in the pathogenesis of DKD. Epigenetic variation mainly includes DNA methylation, histone modification and changes in the noncoding RNA expression profile, which are deeply involved in DKD-related inflammation, oxidative stress, hemodynamics, and the activation of abnormal signaling pathways. Since DKD is reversible at certain disease stages, it is valuable to identify abnormal epigenetic modifications as early diagnosis and treatment targets to prevent the progression of end-stage renal disease (ESRD). Because the current understanding of the epigenetic mechanism of DKD is not comprehensive, the purpose of this review is to summarize the role of epigenetic modification in the occurrence and development of DKD and evaluate the value of epigenetic therapies in DKD.

## Introduction

Diabetic kidney disease (DKD) is a common complication of diabetes and a major cause of end-stage renal disease (ESRD), which seriously affects the quality of life of patients (1–4). The main pathological features of DKD are glomerular sclerosis, podocyte detachment, epithelial-mesenchymal transition (EMT)/endothelial-to-mesenchymal transition (EndMT)/macrophage-myofibroblast transition (MMT), excessive extracellular matrix (ECM) and renal tubular fibrosis. These pathological changes affect glomerular and tubular function, leading to the progression of proteinuria and decreased glomerular filtration capacity. A long-term hyperglycemic environment in diabetics induces metabolic disorders, oxidative stress and hemodynamic changes. Although these symptoms occur with genetic mutations, they are, to a greater extent, related to epigenetic variations (5). For instance, studies have found that even after a long period of strict glycemic control, patients with diabetes may still develop complications due to early high glucose (HG) exposure (4, 6, 7). This metabolic memory phenomenon has been shown to be related to DNA methylation and histone acetylation at the promoter, which suggests that epigenetic modifications are subsumed in the pathological process of diabetes and affect patients’ conditions over a long period of time (8, 9). Therefore, a deeper understanding of the epigenetic modifications in DKD can help to better understand the pathogenesis of the disease and provide potential predictive and therapeutic targets for DKD treatment. In the current study, we conducted a comprehensive analysis and introduction of DKD-related epigenetic mechanisms and epigenetic therapies based on searching the published literature from PubMed (https://pubmed.ncbi.nlm.nih.gov) and Web of Science (http://www.webofknowledge.com/databases ). Our aim is to encourage more clinicians and researchers to pay attention to the function of epigenetic modifications in the occurrence and development of DKD and conduct laboratory, preclinical and clinical studies on the development of epigenetic drugs and therapeutic strategies for DKD.

## The pathogenesis of DKD

**Figure 1:**
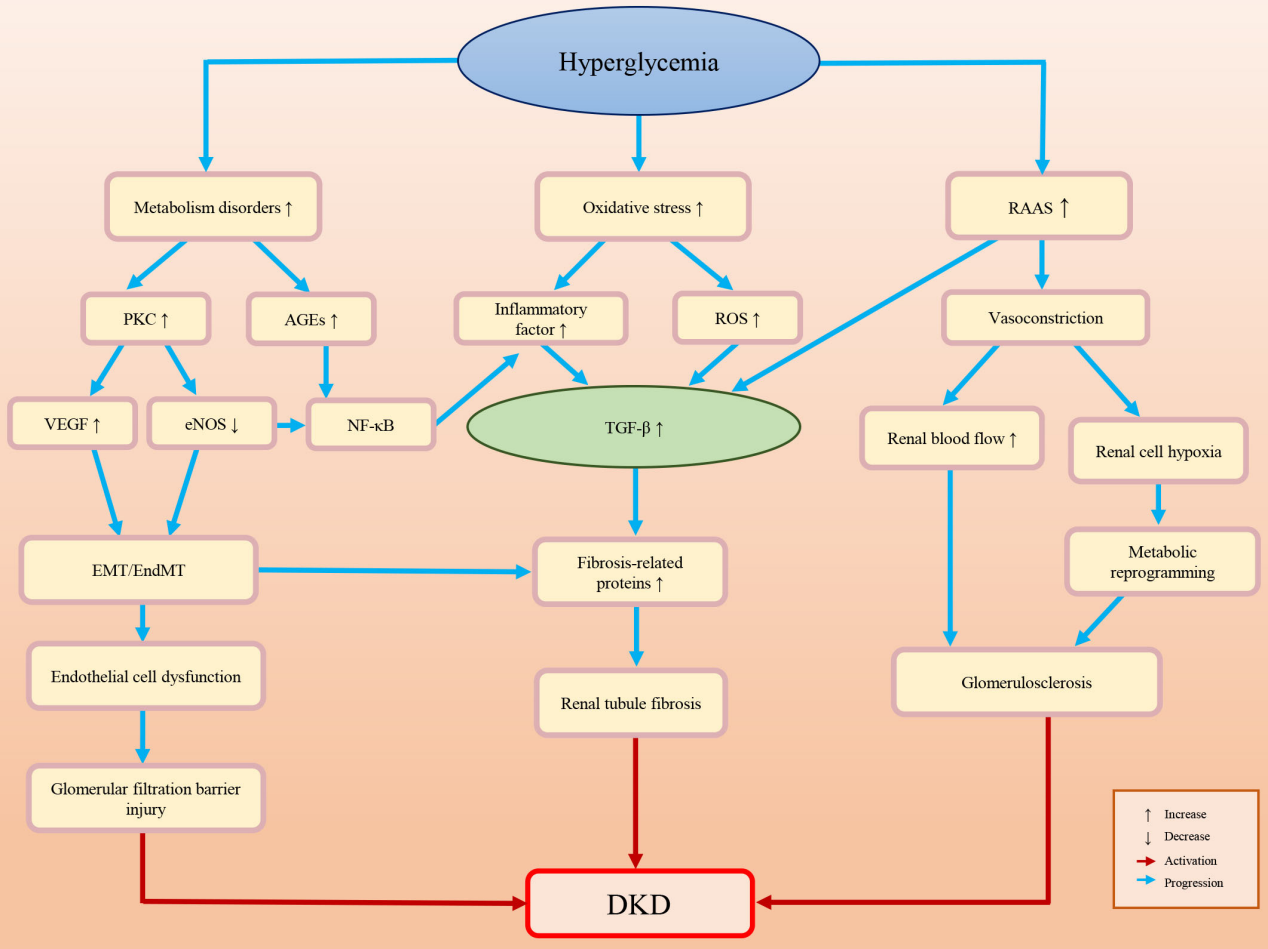
Briefly summarizes and illustration of the pathogenesis of DKD.

Diabetic patients often have high blood pressure, high blood lipids, high uric acid and obesity, all of which may lead to kidney damage (10, 11). The pathogenesis of DKD is complex. The main pathological characteristics of DKD are glomerulosclerosis and renal fibrosis (12, 13). An impaired glomerular filtration barrier is the primary cause of albuminuria. Renal fibrosis and albuminuria are important causes of renal function loss, which is the consequence of multiple factors and mechanisms. DKD-associated renal fibrosis is defined by the excessive deposition of ECM caused by various adverse stimuli (14–17). Understanding the pathogenesis of DKD may help to prevent, slow down, or even reverse DKD. **Figure 1** briefly summarizes the pathogenesis of DKD.

### Metabolism disorders

Glucose, lipid and hormone metabolism disorders caused by HG exposure may lead to the accumulation of advanced glycation end products (AGEs) and the activation of protein kinase C (PKC) (18–22). AGEs can activate related signaling pathways, such as the nuclear factor kappa-B (NF-κB) and transforming growth factor β (TGF-β) pathways, promote EMT/EndMT, and result in glomerular podocyte loss and progressive glomerulosclerosis (23–27). Activated PKC may decrease endothelial nitric oxide synthase (eNOS) production, which not only activates NF-κB-mediated inflammatory pathways but also stimulates the production of vascular endothelial growth factor (VEGF), inducing endothelial dysfunction and further (26, 28–30).

### Oxidative stress

The HG environment activates polyols, PKC, hexosamine and other pathways, leading to an increase in the oxidative stress response and reactive oxygen species (ROS) (31–33). ROS mediate various signaling pathways, such as TGF-β, adenosine 5′ monophosphate-activated protein kinase (AMPK) and nuclear factor-erythroid 2-related factor 2 (Nrf2), which pertain to the cell cycle, cell proliferation, autophagy, inflammation and oxidative stress (33–35). In DKD, the activation of ROS can promote podocyte apoptosis and inflammatory factor release, and activate the renal fibrosis signaling pathways, which results in renal fibrosis and the decline of glomerular filtration function (23, 25, 27).

### Inflammation

Diabetes is often accompanied by chronic inflammation. The expression levels of inflammatory factors (e.g. tumor necrosis factor-α (TNF-α), interleukin-6, interferon γ (IFN-γ) and interleukin-17) are elevated in DKD patients (14–16, 36–41). Abnormal expression of these cytokines may activate renal fibrosis-related signaling pathways, induce EndMT/EMT/MMT, and promote the accumulation of ECM, which ultimately stimulates the expression of fibrosis-related proteins (e.g. α-smooth muscle actin (α-SMA) and connective tissue growth factor (CTGF)) and glomerulosclerosis (42–47).

### Hemodynamic change

Diabetic patients’ kidneys are always exposed to an HG environment for a long time. The long-term high level filtration load may induce glomerular feedback dysfunction and aggravate glomerular sclerosis (48). The renin-angiotensin-aldosterone system (RAAS) can also be activated by HG exposure (e.g., the products of HG-induced metabolic disorders and oxidative stress-induced ROS) (49). The RAAS not only induces the constriction of blood vessels in the kidney, but also upregulates TGF-β fibrosis-associated pathways and inflammation (49–53). The decline in blood flow and oxygen delivery at the glomerular filtration barrier after renal vasoconstriction may promote glycolysis and metabolic reprogramming and produce metabolites (e.g., lactate and L-serine) (54–58). These metabolites are associated with multiple cellular behavior variations, such as mitochondrial damage, histone modification, and the activation of the renal cell fibrosis-related signaling pathway, which may affect cell senescence and survival, increase inflammation reflection, induce podocyte damage, endothelial cell dysfunction, and renal tubular cell fibrosis, and further aggravate kidney damage (59–64).

## The epigenetic modification of DKD

### DNA methylation in DKD

DNA methylation is a significant epigenetic regulatory mechanism. DNA methylation is catalyzed by a family of DNA methyltransferases that transfer a methyl group from S-adenyl methionine to the carbon of a cytosine residue (65). DNA methylation can change chromosome structure, conformation, stability and the interaction mode between DNA and protein, thereby participating in a variety of regulatory mechanisms (e.g., gene transcription and imprinting, cell differentiation and fibrosis) (66–70).

DNA methylation is associated with DKD. VanderJagt et al. found that many methylation modifications occur from prediabetes to diabetes. Among these methylation modifications, six genes are associated with DKD, which may induce inflammation and immunity and break urate homeostasis (71). By comparing the DNA methylation of kidney proximal tubule cells in 10-week-old db/db mice with that in normal mice, Marumo et al. considered that at the early stage of DKD, several potentially functional genes were significantly methylated, e.g., angiotensinogen (*Agt*) and claudin 18 (*Cldn18*), which may alter the progression of DKD (72). Park et al. indicated that there are extensive methylation differences in DKD kidneys, among which the change in TNF-α methylation has a close connection with kidney function decline (73). In addition, the application of reversed-phase high performance liquid chromatography (RP-HPLC) to determine DNA methylation levels in peripheral blood mononuclear cells also revealed differences in genomic methylation levels between patients with renal dysfunction and patients with simple diabetes (74, 75). These abnormal changes may be a response to a chronically hyperglycemic environment. Furthermore, the degree of methylation in DKD varies from stage to stage. Lecamwasam et al. collected blood samples from diabetic patients with chronic kidney disease (CKD) and indicated that differential methylation patterns of 5’-C-phosphate-G-3’ (CpG) sites are associated with different stages of CKD. Of note, relative to the early CKD group, the cysteine-rich secretory protein 2 (*CRISP2*) gene promoter carried 12 hypermethylated CpG sites in the late CKD group, which may lead to oxidative stress in inflammatory pathways (76).

### Histone modification in DKD

**Figure 2:**
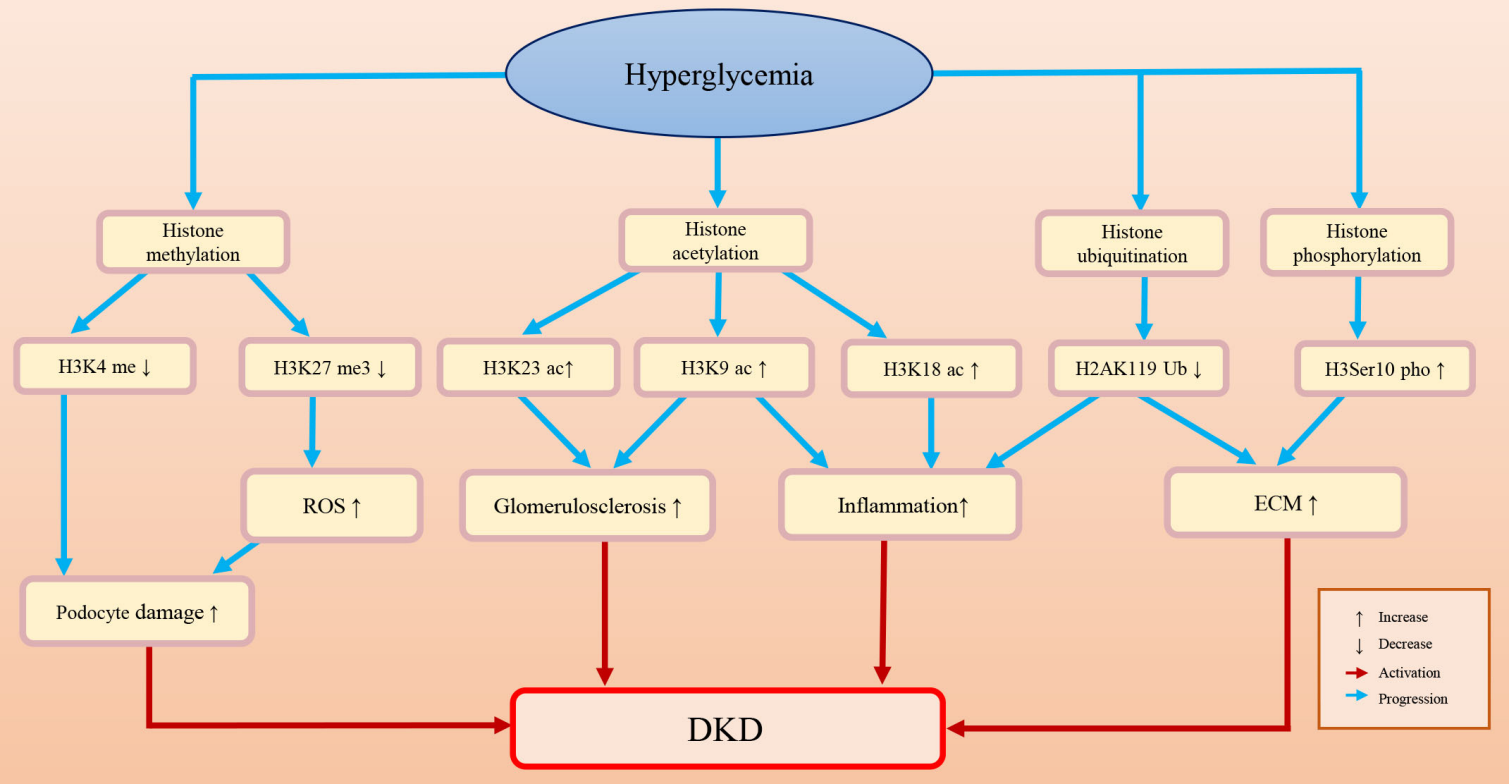
The histone epigenetic modifications and their roles in DKD.

Histones are an important component of nucleosomes and a general term for alkaline proteins that bind to DNA (77–79). The free N-terminus at the end of histones can undergo various modifications, including acetylation, methylation, phosphorylation, and ubiquitination (80). Once histones are modified, the function of chromatin will be changed: first, the charge of amino acids will be changed, and the affinity between histones and DNA will be decreased; second, binding to specific surfaces and regulating transcriptional activity will also be changed (79). **Figure 2** briefly summarizes the histone epigenetic modifications and their regulatory roles in DKD.

#### Histone methylation

Histone methylation is a process in which methyl groups are transferred to lysine and arginine residues in the histone tail by histone methyltransferase (81). Histone methylation is a dynamic and reversible process because the methylation of histones can be erased by histone demethylases (82). Histone methylation plays a regulatory role similar to that of DNA methylation. Whether it functions in transcriptional repression or activation depends on the methylation degree and the modification site.

Histone methylation is an important epigenetic modification in DKD. In diabetic mice, upregulation of TGF-β may promote the recruitment of the histone H3 lysine 4 (H3K4) methylation methyltransferase SET7/9 and upregulate the expression levels of H3K4me and p21. This may lead to glomerular cell injury, severe glomerular sclerosis, albuminuria, and a decreased glomerular filtration rate (83). Histone methylation also affects podocyte survival and function. The foot processes of podocytes are attached to the basement membrane of the glomeruli. Foot process effacement and simplification can lead to proteinuria, which is a sign of podocyte injury (84, 85). Adjacent podocytes connect through the slit diaphragm and form an important barrier for glomerular filtration proteins (86). Therefore, the structure and arrangement of podocytes are very important to kidney function (85). PAX transcription activation domain interacting protein (PTIP) is a part of the H3K4 methyltransferase complex (87). Lefevre et al. found that the H3K4 trimethylation (H3K4me3) level declined in PTIP knockout mouse podocytes, which may affect the transcription of the neurotrophic tyrosine kinase receptor type 3 (*Ntrk3*) gene, resulting in podocyte development disorder and abnormal podocyte arrangement and eventually leading to tubulointerstitial fibrosis and glomerulosclerosis (88, 89). Furthermore, PTIP can interact with dachshund homolog 1 (DACH1) and be recruited by DACH1 to its promoter-binding sites. In podocytes, DACH1-PTIP recruitment can repress transcription, limit promoter H3K4me3, and affect the transcription of downstream genes (89–92). Cao et al. found that DACH1 played a safeguard role in podocytes. DACH1 expression is dramatically decreased in DKD patients, which may result in proteinuria. In DACH1 knockdown podocytes combined with hyperglycemia, DACH1-PTIP promoter binding was reduced, transcriptional repression was lost, and the H3K4me3 expression level was increased (88).

Decreased expression of H3K27me3 in DKD may aggravate podocyte injury and fibrosis. Enhancer of zeste 2 polycomb repressive complex 2 subunit (EZH2), a methyltransferase, can regulate podocyte oxidative stress and renal injury in diabetes (93, 94). DKD patients often have metabolic disorders and high levels of AGEs. Liebisch et al. found that in podocytes of diabetic mice, high levels of AGEs can downregulate EZH2 expression levels, decrease H3K27me3 levels, and induce podocyte injury (95). Siddiqi et al. found that in diabetic rats, depletion of EZH2 may decrease H3K27me3 levels and increase glomerular thioredoxin interacting protein (TxnIP) expression levels, which may promote ROS accumulation, increase matrix production, and lead to podocyte injury and proteinuria (96). Similarly, Ye et al. studied the safeguard role of H3K27me3 and EZH2 in a rat DKD model and indicated that in rat renal mesangial cells, TGF-β downregulated the expression of EZH2, decreased the enrichment of the epigenetic repressive mark H3K27me3 at the fibrotic gene promoter (e.g., Serpin family E member 1 (*Serpine1*) and C-C motif chemokine ligand 2 (*Ccl2*), and increased fibrosis protein expression and renal fibrosis (97). Ubiquitously transcribed tetratricopeptide repeat on chromosome X (UTX) is a demethylase that removes dimethyl and trimethyl groups from H3K27 (98). UTX expression is increased in the renal tubules of diabetic mice and DKD patients (99). Increased UTX may promote the transcription of inflammatory factor genes and DNA damage. However, administration of the H3K27 demethylase inhibitor GSK-J4 alleviated inflammatory damage to renal tubules in diabetic mice (99).

Glucocorticoid receptor (GR) and mineralocorticoid receptor (MR) play pivotal roles in DKD-associated fibrosis and inflammation. GR and MR are expressed in a variety of renal cells (e.g., podocytes, endothelial cells and fibroblasts). The absence of GR may induce renal fibrosis and albuminuria (100, 101). Overactivation of MR may lead to endothelial dysfunction, renal fibrosis, and renal water and salt metabolism dysfunction (102, 103). Water and salt metabolism disorder is a common metabolic abnormality in DKD patients (104). Disturbance of renal water and salt metabolism may lead to sodium retention, blood pressure elevation, glomerular sclerosis, and tubulointerstitial fibrosis (102). The expression levels of GR and MR are closely related to epigenetic modification. Disruptor of telomeric silencing-1 (Dot1) is a histone lysine methyltransferase whose function and activity are regulated by GR (104). When MR is deficient, GR can modify Dot1 methyltransferase activity through the serum/glucocorticoid-regulated kinase 1 (Sgk-1) and aldosterone (a corticosteroid)-dependent signaling pathways, thereby relaxing chromatin in relevant locations and promoting transcription to compensatively increase epithelial sodium channel expression (104–107). In this way, kidney salt retention can be regulated and the filtration function of the kidney can be ensured.

#### Histone acetylation

Histone acetylation usually occurs on lysine residues. Lysine is positively charged, and DNA is negatively charged. Under normal conditions, histone proteins and DNA are tightly bound by interaction. When histone acetylation occurs, acetyl-coenzyme A is transferred to the lysine side chain, which disrupts the interaction between histones and DNA and leads to nucleosome structure relaxation and a subsequent increase in accessibility to transcription factors (108, 109).

Histone acetylation plays an important role in the onset of DKD. Chen et al. found increased acetylation of H3K9 and H3K18 in the renal cortex of diabetic mice, which is related to inflammatory responses and glomerulosclerosis (110). Sufyan et al. found that the increased acetylation of H3K9 and H3K23 is associated with albuminuria and glomerulosclerosis in a mouse model (83). Lizotte et al. found that H3K9/14 acetylation was associated with insulin resistance, podocyte apoptosis and kidney injury (111).

Histone deacetylases (HDACs) are epigenetic regulatory factors that can reverse the histone acetylation process. HDACs can be divided into four groups according to their homology: class I includes HDAC1/2/3/8; class II includes HDAC4/5/6/7/9/10; class III includes sirtuin (SIRT)1-7; and class IV includes HDAC11 (112). Wang et al. found that the expression of HDAC2/4/5 was increased in streptozotocin (STZ)-induced diabetic rats and db/db mice, and the increased expression of HDAC4 exacerbated inflammation and led to podocyte injury (113).

HDAC3, as a profibrotic factor, plays a pivotal role during the genesis of DKD (114). The expression level of HDAC3 is upregulated in renal tubular epithelial cells of DKD mice (115). Klotho protein protects the kidney by regulating the expression of fibrinogen and prevents renal fibrosis by inhibiting profibrotic signaling pathways (e.g., TGF-β/small mothers against decapentaplegic (Smad) and wingless/integrated (Wnt)/β-catenin (115, 116). HDAC3 may modulate the expression of Klotho. Chen et al. found that HDAC3 promotes renal fibrosis by inhibiting the transcription of the antifibrotic protein Klotho (115). HDAC3 also regulates macrophage function, promotes macrophage M2 polarization activation and leads to MMT, which is a marker of renal fibrosis (117, 118). HDAC3 inhibitors can reverse M2 polarization and the phagocytic activity of macrophages and alleviate renal fibrosis (115, 118).

SIRT3 plays a protective role in DKD-related kidney injury. In DKD patients, HG can downregulate SIRT3, which inhibits the activity of antioxidant enzymes, aggravates oxidative stress, induces mitochondrial dysfunction and leads to the accumulation of metabolic substances such as ROS (60, 61). These variations cause a series of changes in kidney cells, including metabolic reprogramming and immunoreaction fibrosis, and eventually induce kidney damage (61, 119–121). Protein Kinase B (AKT) is involved in apoptosis and proliferation by regulating the phosphorylation of forkhead box O (FoxO) (122). High levels of SIRT3 may inhibit the activity of the Akt/FoxO signaling pathway and reduce oxidative stress and renal tubular epithelial cell apoptosis (123). SIRT3 also plays a role in maintaining endothelial cell homeostasis (124). Srivastava et al. reported that SIRT3 is involved in the regulation of EndMT, and SIRT3 deficiency in mouse endothelial cells may induce/aggravate renal fibrosis. However, renal fibrosis can be relieved by the overexpression of SIRT3 (124).

#### Histone ubiquitination

Ubiquitin is a protein with a highly conserved sequence (125). Histone ubiquitination often occurs at specific lysine residues in the C-terminal tails of histone H2A and histone H2B (126). Three enzymes are involved in the process of histone ubiquitination. First, the ubiquitin molecule is activated by E1 (ubiquitin-activating enzyme) in an ATP-dependent manner; then, the activated ubiquitin moiety forms a complex with E2 (ubiquitin-conjugating enzyme) with the assistance of E1, and the complex is transferred to the target protein with the assistance of specific E3 (ubiquitin ligase) (127). The process of ubiquitination is dynamic and reversible, and deubiquitination enzymes can reverse this process (128). The ubiquitin proteasome system is involved in the degradation of many types of proteins, which is associated with the regulation of a series of cell behaviors and the occurrence of diseases (129, 130).

In diabetic patients, ubiquitin A-52 residue ribosomal protein fusion product 1 gene (UbA52), which is associated with renal tubular injury, and the UbA52 expression level can be upregulated in response to increasing concentrations of glucose (131, 132). Abnormal ubiquitination modifications have also been observed in DKD models. Increased H2A ubiquitination and decreased H2B ubiquitination levels have been observed in HG-treated mesangial cells. In addition, these histone ubiquitination changes may enhance the activation of TGF-β and influence the pathogenesis of DKD (126, 133). Histone ubiquitination can regulate the expression of downstream proteins by changing their occupancy in the promoter region and thus promote renal fibrosis. For example, decreased occupancy of H2AK119 monoubiquitination (H2AK119Ub) at the *TGF-β* and monocyte chemoattractant protein-1 (*MCP-1*) promoters may upregulate TGF-β pathway-related factors in diabetic kidneys, activate fibrosis-related signals, and accelerate renal fibrosis (134). Intriguingly, histone methylation has been shown to be cross-regulated by histone ubiquitination. Goru et al. found that in diabetic kidneys, decreased occupancies of H2AK119Ub may increase occupancies of histone H3K36 dimethylation (H3K36me2) marks on the promoter of *SET7/9* and upregulate the protein SET7/9 expression. Of note, the increased expression level of SET7/9 can increase the promoter occupancies of H3K4me2 on the promoter of collagen type I alpha 1 (*COL1A1*), which may lead to ECM deposition in the kidney and renal fibrosis (135).

Currently, ubiquitin proteasome system-related proteasome inhibitors have been approved for cancer therapy with good efficacy. However, studies on histone ubiquitination modification in DKD are few, and related drug development remains in the experimental stage. Aspirin and Carbobenzoxyl-L-leucyl-L-leucyl-L-leucine (MG132) are potential proteasome inhibitors. Aspirin can prevent and alleviate renal fibrosis in diabetic animals by increasing histone H2AK119Ub and reducing SET7 deposition-induced ECM (136). MG132 alleviates oxidative stress-induced damage to the kidney by inhibiting diabetes-increased proteasome activity and upregulating Nrf2 (137). Although these drugs are in the preclinical stage, proteasome inhibitors have shown promising therapeutic potential in DKD treatment.

#### Histone phosphorylation

Histone phosphorylation is a central step in chromosome coagulation, transcriptional regulation, and DNA damage repair during cell division (78, 138, 139). In DKD mice and patients, the increase in histone H3 serine 10 (H3Ser10) phosphorylation may upregulate vascular cell adhesion molecule 1 (VCAM-1), promote glomerular endothelial activation, and activate DKD fibrosis and inflammation progression (26, 140). Histone phosphorylation is associated with albuminuria. Sayyed et al. found that glomerulosclerosis and albuminuria were associated with increased H3Ser10 phosphorylation, and the process of histone phosphorylation could be reversed. Ccl2 blockade can prevent the progression of DKD by blocking H3Ser10 phosphorylation (83). Moreover, Tikoo et al. found that resveratrol (a kind of polyphenol) can prevent kidney disease progression by reducing H3 dephosphorylation in diabetic rats (141, 142).

### NcRNA changes in DKD

#### Long noncoding RNA (LncRNA) and DKD

LncRNAs are a class of RNA molecules whose transcript length exceeds 200 nt (143). Instead of encoding proteins, lncRNAs regulate cell behaviors by influencing gene transcription and protein translation (144). LncRNAs are associated with the occurrence and development of a variety of diseases. In DKD, lncRNAs are involved in renal fibrosis, inflammation, podocyte injury, albuminuria and other pathological processes in direct or indirect ways (145).

LncRNAs are crucial during the genesis of DKD. LncRNAs can affect protein expression by targeting microRNAs (miRNAs) and related signaling pathways. miR-96-5P regulates the expression of fibronectin, which is involved in renal fibrosis. It has been observed that the expression level of miR-96-5P is downregulated in HG-treated renal tubular epithelial cells and DKD mouse models (146). LncRNA GAS5 can bind to miR-96-5p and inhibit its expression, thereby promoting renal fibrosis (146). HG may stimulate the expression of lncRNA NR_038323. In STZ-induced DKD mice, lncRNA NR_038323 may interact with miR-324-3p, which upregulates the expression of dual-specificity protein phosphatase-1 (DUSP1), downregulates the expression of collagen I, collagen IV and fibronectin, and significantly improves renal fibrosis and glomerular hypertrophy (147). In the early stage of DKD, the expression level of lncRNA CYP4B1-PS1-001 is significantly downregulated. However, the enforced expression of lncRNA CYP4B1-PS1-001 can inhibit the proliferation and fibrosis of murine mesangial cells by interacting with nucleolin (148, 149). LncRNA SOX2OT can exert renal protective effects by inhibiting renal fibrosis (150, 151). In DKD mice, overexpression of lncRNA SOX2OT may alleviate hyperglycemia, decrease the expression of fibronectin, suppress collagen-related interstitial fibrosis, enhance the autophagy of mesentery cells, and significantly inhibit the proliferation and fibrosis of mesentery cells (150).

LncRNAs are also associated with proteinuria. The expression of lncENST00000436340 is increased in DKD patients. It has been demonstrated that lncENST00000436340 may promote the degradation of polypyrimidine tract binding protein 1 (PTBP1) by enhancing its binding to mRNA, which regulates cytoskeletal rearrangement, and leads to podocyte injury and urine protein (152). LncRNA DLX6-AS1 is highly expressed in DKD patients and podocytes cultured in HG. cAMP-response element binding protein (CREB) can target DLX6-AS1, and overexpression of CREB may increase the level of DLX6-AS1. High levels of DLX6-AS1 may disrupt the podocyte structure, increase kidney inflammation, and induce albuminuria (153).

#### MiRNA and DKD

miRNAs are a class of small and highly conserved noncoding RNAs that regulate protein expression at the posttranscriptional level by interacting directly with the 3’UTR of target genes (154). miRNAs also participate in the pathogenesis of DKD. It has been demonstrated that the expression of miRNA-5b-181p is decreased in a DKD mouse model, and supplementation with miRNA-5b-181p-mimic may reduce albuminuria and alleviate abnormal mesentery expansion (155). Since miRNA can be stably present in urine in the form of exosomes, it can be used as a biomarker to predict the progression of DKD. It has been reported that the expression of miR-342b, miR-30 and miR-2a is significantly increased in the urinary exosomes of DKD patients (156).

miR-33 and miR-21 play significant roles in renal fibrosis. miR-33 can promote fibrosis by activating the TGF-β/Smad inflammatory pathway (157, 158). In a folate-treated mouse model, miR-33 deletion enhanced fatty acid oxidation, reduced lipid accumulation, and protected mouse kidneys from fibrosis (159). miR-21 expression is upregulated in DKD patients. It has been demonstrated that miR-21 in the exosomes of renal tubular cells can target the phosphatase and tensin homolog (PTEN)/AKT pathway and promote renal fibrosis (160). TGF-β/Smad3 mediates the upregulation of miR-21 in renal tubular epithelial cells, which in turn positively regulates the expression of ECM and α-SMA in TECs and fibrotic kidneys (161). The TGF-β/Smad3 pathway also induces the expression of renal tubule collagen I, promotes ECM accumulation and accelerates renal fibrosis by promoting the expression of miR-192 (162, 163).

Fibroblast growth factor receptor 1 (FGFR1) plays a key role in the anti-EndMT process and in reducing kidney fibrosis (164, 165). Koya et al. performed a series of studies on DKD-related EndMT and found that there was EndMT-related crosstalk between miR-29, miR-let-7 family members and FGFR1 (166–168). Overactivation of the TGF-β/Smad signaling pathway may decrease the expression of miR-29, which promotes the transcription of the inflammatory factor IFN-γ and inhibits FGFR1, leading to a downregulation of FGFR1-dependent miR-let-7 (166, 169, 170). The decreased expression of miR-let-7a enhances glycolysis, increases lactic acid and ROS accumulation, turns on metabolic reprogramming and leads to EndMT (54, 55, 167). Furthermore, N-acetyl-seryl-aspartyl-lysyl-proline (AcSDKP) can maintain endothelial homeostasis and protect the kidney from fibrosis by activating FGFR1 and miR-let-7 (167, 171–173).

#### Circular RNA (CircRNA) and DKD

CircRNAs are a class of single-stranded closed-loop RNAs that mainly exist in the cytoplasm or exosomes. Functionally, circRNAs can interact with proteins and other RNAs by acting as microRNA sponges and regulate transcription in either a competitive or noncompetitive fashion; in some cases, circRNAs can also be translated into polypeptides and perform regulatory functions (174–176).

CircRNA profiles vary with different physiological states, so they can be used as biomarkers and therapeutic targets of diseases. The most common function of circRNAs in DKD is serving as molecular sponges through targeting miRNA and functional proteins, such as SIRT6, SRY-Box Transcription Factor 6 (SOX6), TGF-β1 and NF-κB. CircRNAs are widely involved in DKD-related oxidative stress, inflammation, ECM accumulation and renal fibrosis (177). Qin et al. found that the HG environment can increase the expression levels of circ_0123996 and SOX6 and decrease the expression of miR-203a-3p in mesenchymal cells. Silencing circ_0123996 can suppress cell proliferation and alleviate inflammation and fibrosis (178). Ge et al. found that after exposing mesangial cells to a similar HG environment as in DKD patients, the expression of circ_0000064 was increased (179). Knockdown of circ_0000064 may inhibit the expression of fibrosis-related proteins, such as type I collagen, type IV collagen, and fibronectin (25, 179). **Table 1** summarizes the DKD-related circRNAs. Studies of the function of circRNAs in DKD remain at the animal and cell experimental stages, and to date, no circRNA drug has been approved for the clinical treatment of DKD.

**Table 1:** DKD related circRNA.

Circ RNA	Experimental object	Change	Pathway	Effect	Reference
Circ_0000064	Renal tubular epithelial cells	↑	miR-2-532p↓ ROCK3↑	Oxidative stress↑ Apoptosis↑ Fibrosis↑	(180)
	Human renal mesangial cells	↑	miR-424-5p↓ WNT2B↑	Proliferation↑ Inflammation↑ ECM accumulation↑	(181)
	Mouse mesangial cells	↑	miR-30c-5p↓ Lmp7↑	Oxidative stress↑ Inflammation↑ ECM accumulation↑	(182)
Circ_EIF4G2	NRK-52E cells	↑	miR-218↓ SERBP1↑	Fibrosis↑	(183)
Circ_15698	Mouse mesangial cells	↑	miR-185↓ TGF-β↑	ECM accumulation↑	(177)
Circ_AKT3	Mouse mesangial cells	↓	miR-296-3p↑ E-cadherin↓	Apoptosis↑ ECM accumulation↑	(184)
CircRNA_0000491	Mouse mesangial cells	↑	miR-101b↓ TGFβRI↑	ECM accumulation↑	(185)
	Mouse mesangial cells	↑	miR-455-3p↓ HMBG1↑	Apoptosis↑ Inflammation↑ Oxidative stress↑ Fibrosis↑	(186)
Circ_0037128	Human mesangial cells	↑	miR-17-3p↓ AKT3↑	Proliferation↑ Fibrosis↑	(187)
	Podocytes	↑	miR-31-5p↓ KLF9↑	Podocytes injury↑	(188)
Circ_0080425	Mouse mesangial cells	↑	miR-24-3p↓ FGF11↑	Proliferation↑ Fibrosis↑	(189)
	Human umbilical vein endothelial cells	↑	miR-140-3p↓ FN1↑	Cell dysfunction↑	(190)
CircRNA_010383	Mouse glomerular mesangial cells Mouse tubular epithelial cells	↓	miR-135a↑ TRPC135↓	ECM accumulation↑	(191)
CircRNA_0000309	Podocytes	↓	miR-188-3p↓ GPX4↑	Proliferation↑ Fibrosis↑ Podocytes apoptosis↑	(192)
Circ_HIPK3	Rat mesangial cells	↑	miR-185↓ TGF-β↑ Cyclin D1↑	Proliferation↑	(193)
Circ_0114428	Glomerular mesangial cells	↑	miR-185-5↓ Smad3↑	Proliferation↑ Fibrosis↑ EMT↑	(194)
Circ_ACTR2	Human renal mesangial cells	↑	miR-205-5p↓ HMGA2↑	Proliferation↑ Inflammation↑ ECM accumulation↑ Oxidative stress↑	(195)
Circ_AOK1	Human glomerular epithelial cells	↑	miR-520h↓ Smad3↑	Proliferation↑ Fibrosis↑ EMT↑	(196)
Circ_0123996	Mouse mesangial cells	↑	miR-149-5p↓ Bach1↑	Proliferation↑ Fibrosis↑	(197)
	Human mesangial cells	↑	miR-203a-3p↓ SOX6↑	Proliferation↑ Inflammation↑ Fibrosis↑	(178)
Circ_0068087	Renal tubular epithelial cells	↑	miR-106a-5p↓ ROCK2↑	Apoptosis↑ Inflammation↑ Oxidative stress↑ Fibrosis↑	(198)
Circ_0125310	Mesangial cells	↑	miR-422a↓ IGF1R↑ P38↑	Proliferation↑ Fibrosis↑	(199)
Circ_WBSCR17	Renal tubular epithelial cells	↑	miR-185-5p↓ SOX6↑	Apoptosis↑ Inflammation↑ Fibrosis↑	(200)
Circ_000166	Renal tubular epithelial cells	↑	miR-296↓ SGLT2↑	Fibrosis↑	(201)
Circ_0037128	Renal tubular epithelial cells	↑	miR-497-5p↓ NFAT5↑	Inflammation↑ Oxidative stress↑ Fibrosis↑	(202)
Circ_0003928	Renal tubular epithelial cells	↑	miR-506-3p↓ HDAC4↑	Oxidative stress↑ Apoptosis↑	(203)
Circ_SMAD4	Mouse glomerular mesangial cells	↓	miR-377-3p↑ BMP7↓	Inflammation↑ ECM accumulation↑ Apoptosis↑	(204)
Circ_0123996	Mesangial cells	↑	miR-203a-3p↓ SOX6↑	Proliferation↑ Inflammation↑ Fibrosis↑	(205)
Circ_0008529	Renal tubular epithelial cells	↑	miR-485-5p↓ WNT2B↑	Apoptosis↑ Inflammation↑	(206)
Circ_0000285	Podocytes	↑	miR-654-3p↓ MAPK6↑	Podocytes injury↑	(207)
Circ_LRP6	Mouse glomerular mesangial cells	↑	miR-205↓ HMGB1↑ TLR4↑ NF-κB↑	Proliferation↑ Oxidative stress↑ Inflammation↑ ECM accumulation↑	(208)
Circ_NUP98	Human glomerular mesangial cells	↑	miR-151-3p↓ HMGA2↑	Oxidative stress↑ Inflammation↑ Fibrosis↑	(209)
Circ_HIPK3	Renal tubular epithelial cells	↓	miR-326↑ miR-487a-3p↑ SIRT1↓	Proliferation↓ Apoptosis↑	(210)
Circ_0060077	Renal tubular epithelial cells	↑	miR-145-5p↓ VASN↑	Apoptosis↑ Oxidative stress↑ Inflammation↑ Fibrosis↑	(211)
Circ_TLK1	Human mesangial cells	↑	miR-126-5p↓ miR-204-5p↓ AKT↑ NF-κB↑	Inflammation↑ Oxidative stress↑ ECM accumulation↑	(212)
Circ_FBXW12	Human mesangial cells	↑	miR-31-5p↓ LIN28B↑	Inflammation↑ Oxidative stress↑ ECM accumulation↑	(213)
Circ_0003928	Renal tubular epithelial cells	↑	miR-151-3p↓ Anxa2↑	Apoptosis↑ Inflammation↑	(214)
Circ_0000181	C57BL/6 mice	↑	miR-667-5p↓ NLRC4↑	Inflammation↑	(215)
Circ_LARP4	Mouse mesangial cells	↓	miR-424↑ Bax↓	Apoptosis↑ Fibrosis↑	(216)
Circ_0054633	Human umbilical vein endothelial cells	↑	miR-218↓ ROBO1↑ HO-1↑	Vascular endothelial cell dysfunction↓	(217)
Circ_ITCH	Rat mesangial cells	↓	miR-33a-5p↑ SIRT6↓	Inflammation↑ Fibrosis↑	(218)

↑, upregulation; ↓, downregulation; ROCK, Rho kinase; WNT2B, Wnt family member 2B; Lmp7, large multifunctional protease 7; SERBP1, SERPINE1 mirna binding protein 1; TGFβRI, TGFβ-receptor type I; HMGB1, high mobility group box 1; KLF9, kruppel-like factor 9; FGF11, fibroblast growth factor 11; FN1, fibronectin 1; TRPC135, transient receptor potential cation channel 135; GPX4, glutathione peroxidase 4; HMGA2, high-mobility group AT-hook 2; Bach1, BTB and CNC homology 1; Sox6, SRY-Box Transcription Factor 6; IGF1R, type 1 insulin-like growth factor receptor; SGLT2, sodium-glucose cotransporter 2; NFAT5, nuclear factor of activated T cells 5; HDAC, histone deacetylase; BMP7, bone morphogenetic protein 7; MAPK6, mitogen-activated protein kinase 6; TLR4, toll-like receptor 4; SIRT, sirtuin; VASN, vasorin; LIN28B, Lin-28 homolog B; Anxa2, annexin A2; NLRC4, NOD-like receptor family CARD domain-containing protein 4; ROBO1, roundabout 1; Bax, Bcl-2 associated X protein; HO-1, heme oxygenase‑1.

### DKD therapy

#### Current therapies in DKD

Currently, the main therapeutic strategies for DKD are to alleviate or avoid proteinuria by controlling blood glucose and blood pressure and enhancing renal filtration capacity. Since the direct cause of DKD in diabetic patients is high blood glucose, lowering blood glucose is the priority for controlling the progression of DKD. Some hypoglycemic drugs also have therapeutic effects on renal disorders. For example, SGLT2 inhibitors (e.g., empagliflozin) not only reduce the tubule reabsorption of glucose but also improve the kidney filtration capacity and delay the progression of kidney disease by reducing glomerular pressure and albuminuria (219). Overactivation of the RAAS may trigger glomerular hypertension, which in turn promotes the constriction of bulbar arterioles, damages endothelial cells, and leads to albuminuria. Therefore, the use of antihypertensive drugs can significantly prevent renal dysfunction while maintaining normal blood pressure (220). RAAS inhibitors are widely used drugs for the treatment of DKD and have been proven to be effective in all stages of DKD (220–222). **Table 2** summarizes the main regular drugs for DKD treatment.

**Table 2:** Regular therapies of DKD.

Drug	Drug class	Research category	DKD related outcome	Reference
Dulaglutide Liraglutide	GLP-1 agonist	Approved medication	Urinary albumin/creatinine ratio↓ Albuminuria↓	(223–225)
Dapagliflozin Canagliflozin Empagliflozin	SGLT2 inhibitor	Approved medication	Blood pressure↓ Weight↓ Glomerular pressure↓ GFR↑ Albuminuria↓	(226–229)
Sitagliptin Linagliptin	DPP-4 inhibitor	Approved medication	Blood glucose↓ Oxidative stress↓ Inflammation↓ Glomerular injury↓ Albuminuria↓	(172, 230–234)
Captopril Losartan Telmisartan Irbesartan	ACEI and ARB	Approved medication	Blood pressure↓ GFR↑ Albuminuria↓	(220–222, 235)
Finerenone	Mineralocorticoid (Aldosterone) receptor Antagonists	Approved medication	Renal fibrosis↓ Inflammation↓	(236–239)
Spironolactone	Aldosterone receptor antagonists	Approved medication	Blood pressure↓ Inflammation↓ Albuminuria↓	(240, 241)
Sevelamer	AGEs antagonist (phosphate binders)	Approved medication	Inflammation↓	(242)
Pirfenidone	TGF-β inhibitor	Approved medication	Fibrosis↓	(243, 244)
Ruboxistaurin	PKC inhibitor	Clinical trial	Fibrosis↓ Albuminuria↓	(245, 246)
Atrasentan	ETR antagonist	Clinical trial	Fibrosis↓ Albuminuria↓ Blood pressure↓	(247–250)
AcSDKP	Endogenous peptide	Animal experiment	Fibrosis↓	(168, 172, 234, 251, 252)
Fasudil	ROCK inhibitor	Animal experiment	Inflammation↓ Fibrosis↓ Glomerulosclerosis↓	(253–256)
FPS-ZM1	RAGE inhibition	Animal experiment	Glomerular nephrin↑ Inflammation↓ Fibrosis↓ Podocyte injury ↓	(257)

↑, upregulation; ↓, downregulation; GLP-1, glucagon-like peptide-1; SGLT2, sodium-glucose cotransporter 2; DPP-4, dipeptidyl peptidase 4; ACEI, angiotensin-converting enzyme inhibitor; ARB, angiotensin receptor blocker; AGEs, advanced glycation end products; TGF-β, transforming growth factor β; PKC, protein kinase C; ETR, endothelin receptor; GFR, glomerular filtration rate; FPS-ZM1, 4-chloro-N-cyclohexyl-N-(phenylmethyl)-benzamide; RAGE, receptor for advanced glycation end products.

#### Potential epigenetic therapies in DKD

Presently, studies of epigenetic drugs for DKD mostly remain at the animal experimental stage, and histone acetylation inhibitors are a research hotspot. We summarized the potential epigenetic therapies for DKD in **Table 3**. HDACIs have been widely studied in tumors and approved for the treatment of cutaneous T-cell lymphoma and multiple myeloma. HDACIs also have a protective effect against diabetic kidney damage. For example, HDAC2 expression is increased in diabetic rats, and administration of trichostatin A (TSA) may decrease ECM-related protein and mRNA expression and prevent (262). TSA also inhibits the activity of the class II type of HDAC, which plays a similar role in blocking EMT. Xu et al. found that the expression of HDAC5 was increased in the renal tubules of diabetic mice. After TSA administration, the expression of HDAC5 was decreased and the accumulation of ECM was alleviated (264). Valproate (VPA), sodium butyrate (NaB), and vorinostat are all HDACIs that inhibit class I and II HDACs (265). VPA is a branched short-chain fatty acid that can alleviate the damage to renal tubules in STZ-induced diabetic rats, reduce autophagy and stress, reduce proteinuria, and prevent kidney fibrosis (258, 259, 266). NaB is another branched short-chain fatty acid that can reduce inflammation and oxidative damage and relieve albuminuria in diabetic rats (260, 267). Vorinostat can relieve oxidative stress in STZ-induced diabetic rats, and decrease renal tubular cell proliferation and glomerular matrix production (261, 268).

**Table 3:** Epigenetic therapies of DKD.

Type	Drug	Applications	DKD related research status	Treatment outcomes in DKD	Reference
HDACI	VPA	Approved for use in epilepsy	Animal experiment	Apoptosis↓ Fibrosis↓ Kidney injury↓	(258, 259)
HDACI	NaB	In a clinical trial to treat schizophrenia	Animal experiment	Fibrosis↓ Apoptosis↓ Inflammation↓ DNA damage↓ Albuminuria↓	(260, 261)
HDACI	TSA	Pre-clinical	Animal experiment	Fibrosis↓ Albuminuria↓	(262)
HDACI	Vorinostat	Approved for use in cutaneous T cell lymphoma	Animal experiment	Oxidative stress↓ ECM↓ Albuminuria↓	(261)
HDAC	SIRT3	Pre-clinical	Animal experiment	Oxidative stress↓ Kidney injury↓	(123)
H3K27 demethylase inhibitors	GSK-J4	Pre-clinical	Animal experiment	Inflammation↓ Fibrosis↓ Glomerulosclerosis↓ Albuminuria↓	(263)

↑, upregulation; ↓, downregulation. HDACI, histone deacetylase inhibitor; VPA, valproate; TSA, trichostatin A; SIRT, sirtuin.

Although HDACIs have great potential in the treatment of DKD, their drawbacks, such as adverse effects and poor tolerance, should not be ignored (265, 269, 270). For life-threatening diseases such as cancer, side effects such as nausea, vomiting, and liver toxicity are acceptable. However, whether the application of HDACIs is a good choice for chronic diseases such as DKD should be discussed with great deliberation. In addition, the specificity of HDACIs is poor. Because class I, II and IV HDACs are all dependent on zinc for enzymatic reactions, and most HDACIs target the zinc domain, HDACIs have broad spectrum effects (commonly called pan-HDACIs) (270, 271).

## Conclusion and perspectives

**Figure 3:**
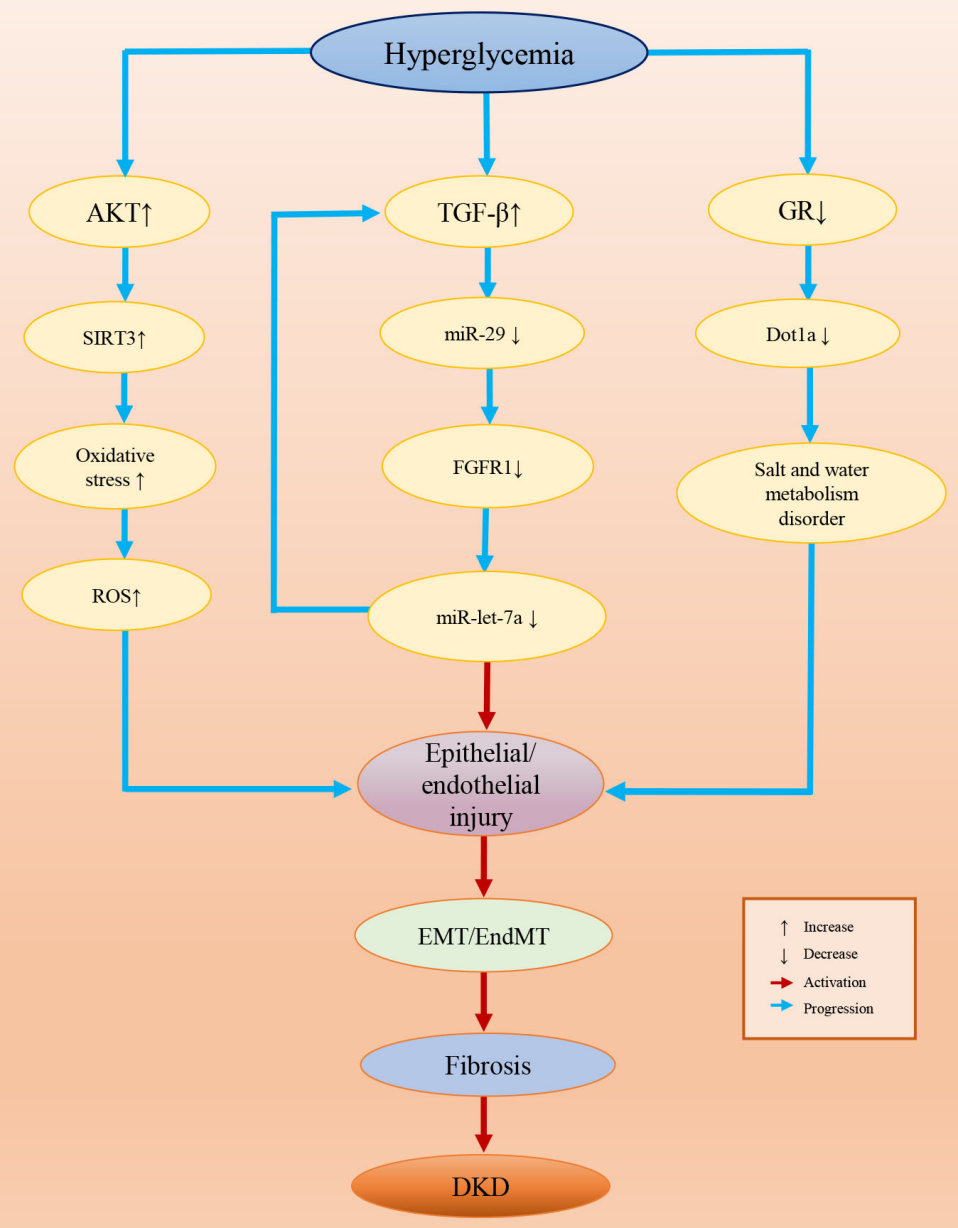
Epigenetic variation-induced EndMT/EMT processes in DKD.

Epigenetic modifications are common in diseases and some epigenetic variations are highly specific in a certain disease or a certain stage of disease, which provides us with potential therapeutic targets in clinical treatments (272, 273). Presently, many studies have confirmed the role of epigenetics in DKD. In this review, we concluded the evidence for epigenetic modifications associated with DKD by summarizing the relevant literature, and we found that epigenetic modifications are involved in the inhibition/activation of a variety of pathogenic signaling pathways. Epigenetic variations affect multiple renal cell functions, such as the activity of GR and glucose metabolism (274, 275). In particular, epigenetic variation-induced EndMT/EMT processes are pivotal in the genesis of DKD, which are the core events in kidney fibrosis (**Figure 3**). Epigenetic modifications are a consequence of exposure to HG and contribute to the progression of DKD. Since DKD is the result of multiple factors and their complex interactions, different epigenetic modifications may contribute to the same outcome through different signaling pathways and mechanisms. However, most of the existing epigenetic studies have focused on the effect of a single variation on the changes in the signaling pathway to promote or mitigate the occurrence of DKD processes. Therefore, drugs or biomarkers designed for a single target are probably not accurate, and the joint use of multiple epigenetic drugs targeting different epigenetic variations should be considered in future DKD treatment. In addition, most of these studies were conducted in diabetic animals or cell models under HG conditions, but we believe that the human body environment is more complex and that more influential factors and mechanisms should be involved in DKD than animals and cells. Therefore, additional solid experimental and clinical trial data from clinical specimens and patients are eagerly anticipated.

In recent years, epigenetic detection technology has developed rapidly. With the wide application of high-throughput sequencing technology in the clinic, the detection of epigenetic changes (mainly DNA methylation and noncoding RNA profiles) in kidney tissues or the peripheral blood of DKD patients has become easier, faster and cheaper to implement (276, 277). These sequencing results are of great value for the precision diagnosis and drug development of DKD. Moreover, the CRISPR−Cas9 system is being tried as a novel tool for editing a specific epigenetic variation, which is a potential approach for the prevention and treatment of DKD (276, 278, 279).

## Author contributions

WS and DY conceived the manuscript. ZL and DY drafted the manuscript. ZL drew the figures. ZL and WW proofread the manuscript and made revisions. LL and XA collected the references. All authors contributed to the article and approved the submitted version.

## Glossary

**Table:**

Abbreviation	Full name
**DKD**	diabetic kidney disease
**ESRD**	end-stage renal disease
**EMT**	epithelial-mesenchymal transition
**EndMT**	endothelial-to-mesenchymal transition
**MMT**	macrophage-myofibroblast transition
**HG**	high glucose
**ECM**	extracellular matrix
**AGEs**	advanced glycation end products
**PKC**	protein kinase C
**NF-κB**	nuclear factor kappa-B
**TGF-β**	transforming growth factor β
**eNOS**	endothelial nitric oxide synthase
**VEGF**	vascular endothelial growth factor
**ROS**	reactive oxygen species
**AMPK**	adenosine 5′ monophosphate-activated protein kinase
**Nrf2**	nuclear factor-erythroid 2-related factor 2
**TNF-α**	tumor necrosis factor-α
**IFN-γ**	interferon γ
**α-SMA**	α-smooth muscle actin
**CTGF**	connective tissue growth factor
**RAAS**	renin-angiotensin-aldosterone system
**Agt**	angiotensinogen
**Cldn18**	claudin 18
**RP-HPLC**	reversed-phase high performance liquid chromatography
**CKD**	chronic kidney disease
**CpG**	5'-C-phosphate-G-3'
**CRISP2**	cysteine-rich secretory protein 2
**PTIP**	PAX transcription activation domain interacting protein
**DACH1**	dachshund homolog 1
**Ntrk3**	neurotrophic tyrosine kinase receptor type 3
**EZH2**	enhancer of zeste 2 polycomb repressive complex 2 subunit
**TxnIP**	thioredoxin interacting protein
**Serpine1**	serpin family E member 1
**Ccl2**	C-C motif chemokine ligand 2
**UTX**	ubiquitously transcribed tetratricopeptide repeat on chromosome x
**GR**	glucocorticoid receptor
**MR**	mineralocorticoid receptor
**Dot1**	Disruptor of telomeric silencing-1
**Sgk-1**	serum/glucocorticoid-regulated kinase 1
**H3K **	histone H3 lysine
**me3**	trimethylation
**me2**	dimethylation
**SIRT**	sirtuin
**HDAC**	histone deacetylase
**STZ**	streptozotocin
**Smad**	small mothers against decapentaplegic
**Wnt**	wingless/Integrated
**AKT**	protein Kinase B
**FoxO**	forkhead box O
**UbA52**	ubiquitin A-52 residue ribosomal protein fusion product 1 gene
**Ub**	monoubiquitination
**MCP-1**	monocyte chemoattractant protein
**COL1A1**	collagen type I alpha 1
**MG132**	carbobenzoxyl-L-leucyl-L-leucyl-L-leucine
**VCAM-1**	vascular cell adhesion molecule 1
**Ser**	serine
**DUSP1**	dual-specificity protein phosphatase-1
**PTBP1**	polypyrimidine tract binding protein 1
**CREB**	cAMP-response element binding protein
**PTEN**	phosphatase and tensin homolog
**FGFR1**	fibroblast growth factor receptor 1
**AcSDKP**	N-acetyl-seryl-aspartyl-lysyl-proline
**SOX6**	SRY-Box Transcription Factor 6
**SGLT2**	sodium-glucose cotransporter 2
**TSA**	trichostatin A
**VPA**	valproate
**NaB**	sodium butyrate
**ROCK**	Rho kinase
**WNT2B**	Wnt family member 2B
**Lmp7**	large multifunctional protease 7
**SERBP1**	SERPINE1 mirna binding protein 1
**TGFβRI**	TGFβ-receptor type I
**KLF9**	kruppel-like factor 9
**FGF11**	fibroblast growth factor 11
**FN1**	fibronectin 1
**TRPC135**	transient receptor potential cation channel 135
**GPX4**	glutathione peroxidase 4
**HMGA2**	high-mobility group AT-hook 2
**Bach1**	BTB and CNC homology 1
**IGF1R**	type 1 insulin-like growth factor receptor
**NFAT5**	nuclear factor of activated T cells 5
**BMP7**	bone morphogenetic protein 7
**MAPK6**	mitogen-activated protein kinase 6
**HMGB1**	high mobility group box 1
**TLR4**	toll-like receptor 4
**VASN**	vasorin
**LIN28B**	Lin-28 homolog B
**Anxa2**	annexin A2
**NLRC4**	NOD-like receptor family CARD domain-containing protein 4
**ROBO1**	roundabout 1
**HO-1**	heme oxygenase−1
**Bax**	Bcl-2 associated X protein
**GLP-1**	glucagon-like peptide-1
**DPP-4**	dipeptidyl peptidase 4
**ACEI**	angiotensin-converting enzyme inhibitor
**ARB**	angiotensin receptor blocker
**ETR**	endothelin receptor
**GFR**	glomerular filtration rate
**FPS-ZM1**	4-chloro-N-cyclohexyl-N-(phenylmethyl)-benzamide
**RAGE**	receptor for advanced glycation end products
**lncRNA**	long noncoding RNA
**miRNA**	microRNA
**circRNA**	circular RNA
